# Mapping information regarding the work-related disability of depression and long-term musculoskeletal pain to the International Classification of Functioning, Disability and Health and ICF Core Sets

**DOI:** 10.3389/fresc.2023.1159208

**Published:** 2023-05-02

**Authors:** Magdalena Fresk, Wilhelmus J. A Grooten, Nina Brodin, Lars G. Backlund, Britt Arrelöv, Ylva Skånér, Anna Kiessling

**Affiliations:** ^1^ Department of Neurobiology, Care Sciences and Society, Division of Family Medicine and Primary Care, Karolinska Institutet, Huddinge, Sweden; ^2^ Department of Neurobiology, Care Sciences and Society, Division of Physiotherapy, Karolinska Institutet, Huddinge, Sweden; ^3^ Women's Health and Allied Health Professionals Theme, Medical Unit Occupational Therapy and Physiotherapy, Karolinska Institutet Hospital, Stockholm, Sweden; ^4^ Department of Orthopaedics, Division of Physiotherapy, Danderyd University Hospital, Stockholm, Sweden; ^5^ Department of Clinical Sciences, Danderyds Hospital, Karolinska Institutet, Stockholm, Sweden

**Keywords:** disability evaluation (MeSH), sick leave certificate, musculoskeletal pain (MeSH), depression, ICF (international classification of functioning disability and health), work capacity evaluation, functional status (MeSH)

## Abstract

**Introduction:**

The International Classification of Functioning, Disability and Health is the WHO coding scheme for functioning-related data. Clear and unambiguous information regarding patients' work-related disabilities is important not only for the assessment of entitlement to paid sickness benefits but also for planning rehabilitation and return to work. The objective was to validate the content of ICF and ICF Core Sets for information on work-related disability in sick leave due to depression and long-term musculoskeletal pain. Specific aims: To describe to what extent (1) such data could be linked to ICF and (2) the result of the ICF linking in terms of ICF categories was represented in relevant ICF Core Sets.

**Methods:**

An ICF-linking study following the ICF-linking rules. A random sample of sick leave certificates issued in primary care for either depression (*n* = 25) or long-term musculoskeletal pain (*n* = 34) was collected from a community with 55,000 inhabitants in Stockholm County, Sweden.

**Results:**

The results of the ICF linking consisted of codings for (1) ICF categories and (2) other health information not possible to link to ICF. The ICF categories were compared to ICF Core Sets for coverage. The majority of the meaning units, 83% for depression and 75% for long-term musculoskeletal pain, were linked to ICF categories. The Comprehensive ICF Core Set for depression covered 14/16 (88%) of the ICF categories derived from the ICF linking. The corresponding figures were lower for both the Brief ICF Core Set for depression 7/16 (44%) and ICF Core Set for disability evaluation in social security 12/20 (60%).

**Conclusion:**

The results indicates that ICF is a feasible code scheme for categorising information on work-related disability in sick leave certificates for depression and long-term musculoskeletal pain. As expected, the Comprehensive ICF Core Set for depression covered the ICF categories derived from the certificates for depression to a high degree. However, the results indicate that (1) sleep- and memory functions should be added to the Brief ICF Core Set for depression, and (2) energy-, attention- and sleep functions should be added to the ICF Core Set for disability evaluation in social security when used in this context.

## 1. Introduction

**Figure 1:**
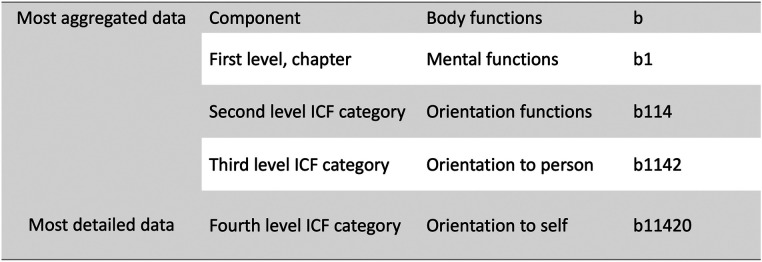
Illustration of the hierarchical structure of the international classification of functioning disability and health, chapter 1 “Mental functions”.

Medical statements of the inability to perform work due to sickness are essential for decisions regarding sickness cash benefit across Europe (1, 2). Clear and unambiguous information regarding patients' work-related disabilities is important not only for the assessment of entitlement to paid sickness benefits but also for planning rehabilitation and return to work (3). In Sweden, sick leave is mainly handled in primary care, and the most common diagnoses are mental disorders (44.8% in Dec 2020) and diseases of the musculoskeletal system (15.9% in Dec 2020) (4, 5). After one week of self-certification, a sick leave certificate issued by a physician is needed (6). Among the information required are the patient's diagnosis and disabilities (impairments of body functions and activity limitations) caused by the disease and related to work. Information on disabilities is structured in accordance with the International Classification of Functioning, Disability and Health (ICF) (7). ICF is offered by the World Health Organization (WHO) as a classification for health data in a perspective wider than the diagnosis alone and contains just above 1,500 ICF categories. ICF has two parts: 1) “Functioning and disability” with the components of *body functions, body structures, activity and participation*; and 2) “Contextual factors” with *environmental factors*. The last component *personal factors* is not yet finalised. Chapters and levels form the hierarchy (Figure 1). In ICF, the term functioning is used for the neutral or positive aspects between a person's health condition and the person's contextual factors, and disability is a term for the negative aspects. A recent international survey by the WHO classification network (WHO-FIC) shows that the use cases for ICF are mainly within clinical practice, but the classification is also used within policy development and education (8). Although the classification is used worldwide, there is a significant variation in the implementation between countries (8–10).

Several tools are developed to support the implementation of the classification, among them ICF Core Sets - selections of ICF categories for diagnoses or specific situations (11). ICF Core Sets are often available in two versions: The comprehensive ICF Core Set, which includes ICF categories for all relevant aspects of functioning of a specific diagnosis, and the shorter brief ICF Core Set with ICF categories for the most central aspects (11). Until now, more than 80 ICF Core Sets have been published, among them, the ICF Core Set for depression, not yet validated in a clinical context, and the ICF Core Set for disability evaluation in social security, designed for the assessment of disability claims and validated in a clinical setting (12, 13).

Data on functional status is needed for social insurance systems but also for public health in general, equality and gender aspects of health, as well as health services planning. The implementation of ICF and ICF Core Sets are ongoing in different settings and countries and needs to be accompanied by validations and analyses of the strengths and weaknesses of the classification in various contexts (8–10). ICF was adopted early as a model for structuring information on work-related disability (impairments of body functions and activity limitations) in sick leave certificates in Sweden, but the content of ICF and ICF Core Sets has not yet been evaluated in relation to information on work-related disability in sick leave certificates.

The overall aim of this study is to validate the content of ICF and ICF Core Sets for health data on work-related disability due to depression and long-term musculoskeletal pain by mapping such information to ICF. The more specific aims were as follows: (1) To describe to what extent the information regarding work-related disability in sick leave certificates could be linked to ICF and (2) to determine to what extent ICF categories derived from the sick leave certificates were represented in relevant ICF Core Sets.

## 2. Material and methods

### 2.1. Design

A standardised procedure of qualitative ICF linking was used to map information on work-related disability in sick leave certificates to ICF (14–16). The comparative validity of ICF Core Sets was assessed by mapping the result of the ICF linking, in terms of ICF categories, to ICF Core Sets.

### 2.2. Ethics

All data were pseudonymised and handled following the ethical approval given by the regional ethical review board of Stockholm, Sweden, Dnr: 2011/1872-31/5.

### 2.3. Setting

Data were collected from a community 60 km northeast of Stockholm, Sweden. The population size was around 55,000, about 50% of whom were aged 20 to 64 years (17). Approximately 10% of the inhabitants were first-generation immigrants, the median income was close to the average national level and the education level was slightly lower than average (18). During the study period, the area had six primary healthcare centres and one local hospital.

### 2.4. Data collection and study sample

Data was originally collected in a quality assurance study based on 177 randomly selected certificates registered between 1 January and 15 August 2012 at the local Social Security Insurance Agency. Inclusion criteria were the following: Sickness certificates issued in primary care and for a sick leave episode of 14 days or longer. If more than one sickness certificate was issued for the same patient during a sickness period, only the most recent was included. Musculoskeletal diagnoses were the main reason for sick leave in 33% of the certificates, along with mental diagnoses in 31% and other diagnoses in 36%. For the current study, three of the authors read all certificates and included those where the main reason for sick leave was either depression or a musculoskeletal diagnosis with long-term musculoskeletal pain described as the main problem (Figure 1). Certificates related to pregnancy were excluded. A total of 25 certificates issued for depression and 34 certificates issued for long-term musculoskeletal pain were included in the study (Table 1).

**Table 1:** Sickness certificates selected for depression and long-term musculoskeletal pain coded with ICD-10-SE and the Swedish national primary care classification KSH97P.

ICD-10-SE	KSH97P	Title	Number of certificates with the diagnosis
Sample depression (*n* = 25)			
F32		Depressive episode	15
F32.9		Depressive episode, unspecified	1
F33		Recurrent depressive disorder	3
F41.1		Generalized anxiety disorder	1
F41.2		Mixed anxiety and depressive disorder	1
F43.9		Reaction to severe stress, unspecified	1
F45.4		Persistent somatoform pain disorder	1
G47		Sleep disorders	1
	F41.9P	Anxiety disorder, other specified	1
Sample long-term musculoskeletal pain (*n* = 34)			
I48		Atrial fibrillation and flutter	1
M16		Coxarthrosis [arthrosis of hip]	1
M18.0		Primary arthrosis of first carpometacarpal joints, bilateral	1
M20.1		Hallux valgus (acquired)	1
M25.5		Pain in joint	3
M47		Spondylosis	1
M47.8		Other spondylosis	1
M51		Other intervertebral disc disorders	1
M51.1		Lumbar and other intervertebral disc disorders with radiculopathy	1
M53.1		Cervicobrachial syndrome	2
M53.3		Sacrococcygeal disorders, not elsewhere classified	1
M54.2		Cervicalgia	1
M54.4		Lumbago with sciatica	2
M54.5		Low back pain	1
M75.1		Rotator cuff syndrome	1
M79.0		Rheumatism, unspecified	3
M79.1		Myalgia	4
R52		Pain, not elsewhere classified	3
	M06P	Rheumatoid arthritis	1
	M53.9P	Dorsopathy, unspecified	1
	M77.1P	Epicondylitis	1
	Q79P	Congenital malformations of the musculoskeletal system, not elsewhere classified (Ehler Danlos syndrome)	1
		Cervical disc disorder, unspecified**	1

Number of certificates wit the diagnosis*

* If more than one code was assigned in the certificate, only the first code is presented in the table.

** No code was assigned, and the diagnosis that was given in free text is presented.

### 2.5. ICF linking of health data

The ICF linking of the information in sick leave certificates was performed by one general practitioner and two physiotherapists, all with expert knowledge of the ICF framework and previous articles in the field. The first five certificates were linked by the group together and the remaining by at least two of the three group members. All items with any kind of inter-rater disagreement were discussed in the full group until a consensus was reached.

The linking procedure followed the updated and refined ICF linking rules as described by Cieza et al. (14–16). First, information in the fields for reporting disabilities was transferred verbatim to an ICF linking protocol. The information was then prepared by separating it into meaning units, i.e., “words, sentences or paragraphs containing aspects related to each other through their own content and context” (19), such as “difficulties walking due to back pain”. The main concept was identified by asking what the piece of health information was about (e.g., “problems with walking”). If the concept could be found in ICF, it was coded with the most precise ICF category (d450 Walk). Any additional concepts (e.g., “pain in the back”) were also identified and coded (b28013 Pain in the back). If the information was insufficient for assigning an ICF category, the concept was coded as not definable (nd) with the additions: general health (gh), mental health (mh), or physical health (ph). If the concept was not included in the ICF, it was coded as not covered (nc), with the addition of hc for health conditions.

A case was handled, but not specifically mentioned in the ICF linking rules, when the context did not explain whether a concept described a functional impairment or an activity limitation as in “the patient has problems with attention”. Such information was linked to both applicable components, i.e., “b140 Attention functions” and “d160 Focusing attention”. It was not annotated if the meaning units described the claimants' preserved functioning or impairments of body functions and activity limitations (disabilities). Therefore, the term functioning is used in this paper as a neutral term and does not indicate a positive or negative aspect of health.

### 2.6. Mapping the ICF categories of functioning derived from sick leave certificates to ICF Core Sets

ICF categories of body functions, and activity and participation, were ranked according to relevance and mapped to the ICF Core Set. Inspired by a previous study in the field, the relevance ranking of the ICF categories was determined to be based on relative frequency across all certificates, that is, the number of certificates containing the ICF category at least one time, out of all certificates (20). The ICF categories above a relative frequency threshold of 10% were chosen for the comparison. ICF categories derived from certificates for depression were compared to the Comprehensive and Brief ICF Core Set for depression (11). ICF Core Set for disability evaluation in social security, a generic ICF Core Set designed for functional assessments in disability benefit claims, was used for certificates issued for long-term musculoskeletal pain since it was found to be the most relevant ICF Core Set for the context, in the absence of a diagnosis-specific ICF Core Set (13).

### 2.7. Main outcome measures

The quantitative results of the ICF linking procedure consisted of 1) codings for ICF categories and 2) other health information not possible to link to ICF. The term codings refers to all codes assigned in the ICF linking process, i.e., all endorsed ICF categories and codes for those not definable and not covered.

### 2.8. Data analyses

The ICF categories were identified according to the ICF linking rules (14–16). The four last certificates were analysed to determine the saturation of ICF categories assumed to be reached if no new ICF category appeared compared to those already linked. The results of the linking in terms of codings were presented per diagnosis. The codings were presented as (1) ICF categories and (2) health data not appropriately codable with ICF. The distribution of all endorsed ICF categories within the components of ICF was described, as well as the granularity (second level or more detailed). Finally, after subtracting duplicates, ICF categories of the components of body functions and activity and participation were aggregated to the second level and mapped to the ICF Core Set.

## 3. Results

### 3.1. Depression

#### 3.1.1. Overall codings

The information was separated into 335 meaning units. A total of 335 main and 39 additional concepts were identified, and these generated 374 codings (Table 2).

**Table 2:** Result of the ICF linking process of health data on work-related disability in sick leave certificates issued for depression and long-term musculoskeletal pain.

	Depression	Long-term musculoskeletal pain
Certificates, *n*	25	34
Meaning units, *n*	335	620
**Identified concepts**		
Main concepts, total, *n*	335	620
Additional concepts, *n*	39	48
Total	374	668
**Codings assigned on the concepts in the ICF linking process**		
*All codings of main and additional concepts, n* (%)		
ICF (all levels)	311 (83)	500 (75)
Not codable with ICF (*not definable*, *not covered*)	63 (17)	168 (25)
Total	374 (100)	668 (100)
*Codings per ICF component, n* (%)		
Body functions	199 (64)	259 (52)
Body structures	3 (1)	34 (7)
Activity and participation	73 (23)	155 (31)
Environmental factors	36 (12)	52 (10)
Total	311 (100)	500 (100)
*Codings of “not codable with ICF”, n* (%)		
Not definable	35 (56)	112 (67)
Not covered	28 (44)	56 (33)
Total	63 (100)	168 (100)
**ICF categories assigned in the ICF linking process**		
*All ICF categories assigned, n* (%)		
Second level	38 (60)	74 (62)
Third level or more granular	25 (40)	45 (38)
Total	63 (100)	119 (100)
*Unique ICF categories on second level per ICF component, n* (%)		
Body functions	25 (48.1)	24 (36.9)
Body structure	3 (5.7)	7 (10.8)
Activity and participation	19 (36.5)	24 (36.9)
Environmental factors	5 (9.6)	10 (15.4)
Total	52 (100)	65 (100)

**Figure 2:**
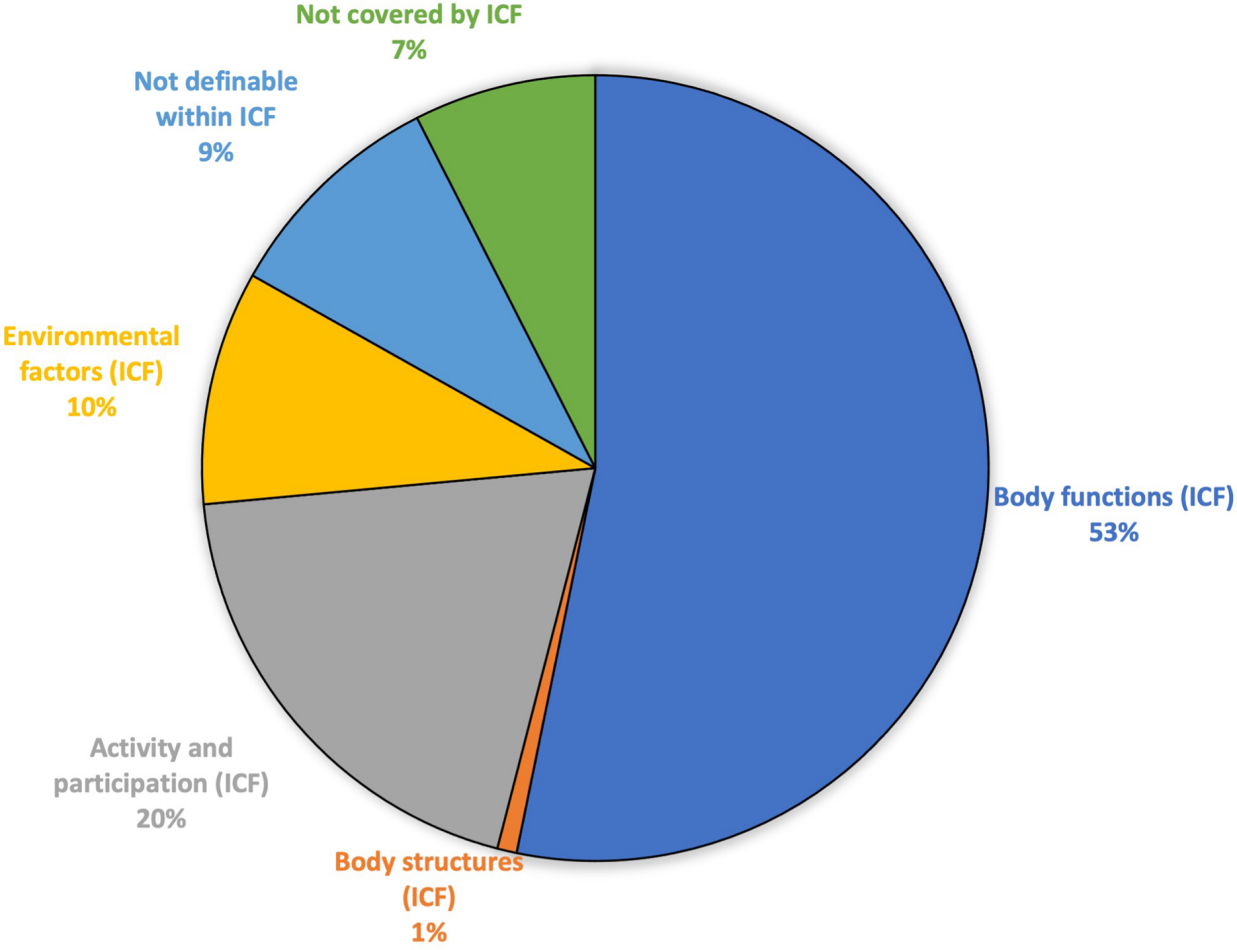
Result of the ICF linking of sick leave certificates issued for depression, in terms of the number of codings of ICF categories and other health information not possible to link to ICF.

The vast majority (311/374, 83%) of the codings were within ICF, and only 63 out of 374 (17%) were not codable by ICF (not definable or not covered) (Figure 2). The codings within ICF were mainly distributed in body functions (199/311, 64%), and activity and participation (73/311, 23%) (Table 2).

Concepts that were not possible to link to ICF contained a variety of information, such as diagnoses, background information, changes in health status, and information regarding measures of assessments, results of assessments, and treatment.

#### 3.1.2. Endorsed ICF categories

The information was linked to 63 different ICF categories, the majority (*n* = 38) on the second level (Table 2). All 25 certificates had information linkable to ICF categories regarding body functions, and 21 of the certificates had at least one ICF category of activity and participation. The concept “focusing on” was in most cases (15 out of 16) linked to both “b140 Attention functions” and “d160 Focusing attention”. Saturation of ICF categories was reached. When ICF categories were aggregated to the second level and duplicates were removed, what remained comprised 25 unique ICF categories for body functions, 19 for activity and participation, five for environmental factors, and three for body structures.

#### 3.1.3. Mapping ICF categories to the ICF Core Set for depression

Ten of the unique second level ICF categories for body functions and six for activity and participation were mapped to the ICF Core Set (Table 3). Nine out of ten (90%) ICF categories regarding body functions were identified in the Comprehensive ICF Core Set, in addition to five out of ten (50%) in the Brief ICF Core Set. For activity and participation, the corresponding figures were five out of six (83%) in the Comprehensive ICF Core Set and two out of six (33%) in the Brief ICF Core Set.

**Table 3:** ICF categories derived from sick leave certificates issued for depression, ranked after relative frequency, and mapped to comprehensive and brief ICF core sets for depression. .

Sickness certificates containing the ICF category ≥ 1 time, *n* (%)*	ICF category***		Comprehensive ICF Core Set	Brief ICF Core Set
**Body functions (Comprehensive *n* = 17** ** **, Brief *n* = 5** ** **)**				
24 (96)	b152	Emotional functions	x	x
18 (72)	b126	Temperament and personality functions	x	x
14 (56)	b130	Energy and drive functions	x	x
11 (44)	b140	Attention functions	x	x
9 (36)	b134	Sleep functions	x	
7 (28)	b144	Memory functions	x	
4 (16)	b164	Higher-level cognitive functions	x	
4 (16)	b410	Heart functions		
3 (12)	b147	Psychomotor functions	x	x
3 (12)	b160	Thought functions	x	
**Activity and Participation (Comprehensive *n* = 45** ** **, Brief *n* = 11** ** **)**				
10 (40)	d160	Focusing attention		
10 (40)	d850	Remunerative employment	x	
8 (32)	d240	Handling stress and other psychological demands	x	x
4 (16)	d710	Basic interpersonal interactions	x	
3 (12)	d210	Undertaking a single task	x	
3 (12)	d230	Carrying out daily routine	x	x

* Relative frequency. The number of certificates containing the ICF category divided by all sickness certificates issued for depression (*n *= 25).

** *n = *total number of categories on the second level in the ICF Core Set for depression per component.

*** The ICF categories are aggregated to the second level, and only those categories present in more than 10% of the sickness certificates are listed.

### 3.2. Long-term musculoskeletal pain

#### 3.2.1. Overall codings

**Figure 3:**
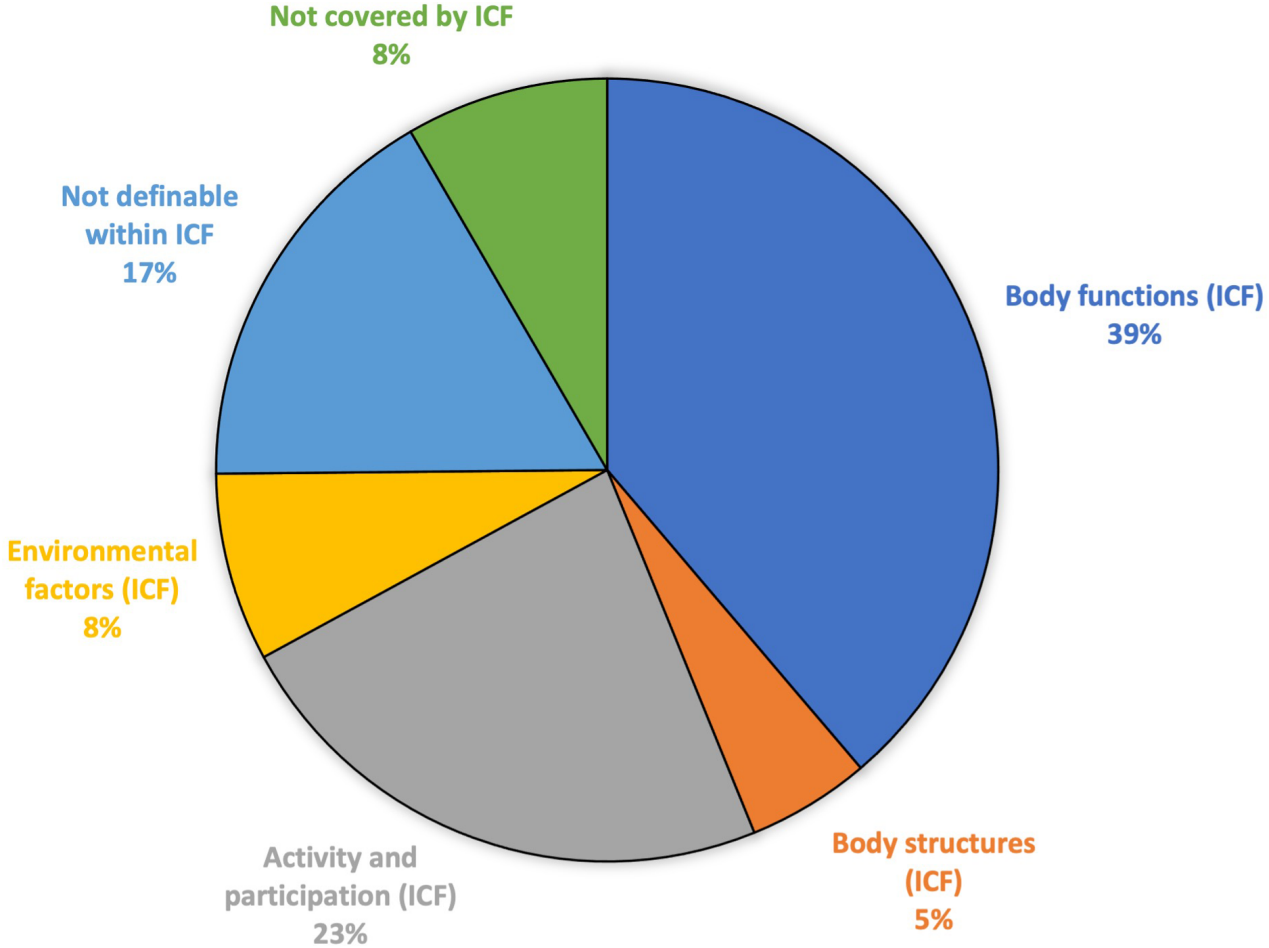
Result of the ICF linking of sick leave certificates issued for long-term musculoskeletal pain, in terms of the number of codings of ICF categories and other health information not possible to link to ICF.

The information in the certificates was separated into 620 meaning units. A total of 620 main and 48 additional concepts were identified and generated 668 codings (Table 2). The vast majority (500/668, 75%) of the codings were within ICF (Figure 3). Twenty-five percent of the codings (168/668) were either not definable with, or not covered by ICF, and the concepts behind these codings were essentially the same as in the certificates for depression. The codings within ICF were distributed in all components, but mainly in body functions (259/500, 52%) and activity and participation (155/500, 31%) (Table 2).

#### 3.2.2. Endorsed ICF categories

The information was linked to 119 different ICF categories, the majority (*n* = 74) on the second level (Table 2). All 34 certificates had information linkable to ICF categories regarding body functions. All but two certificates also included at least one ICF category of activity and participation. Saturation of ICF categories was reached. When the ICF categories were aggregated to the second level and all duplicates removed, 24 unique ICF categories of both body functions, and activity and participation, remained. Also, there were ten unique ICF categories for environmental factors and seven for body structures.

#### 3.2.3. Mapping ICF categories to the ICF Core Set for disability evaluation in social security

Eleven unique ICF categories for body functions, and nine for activity and participation, were mapped to ICF Core Set for disability evaluation in social security (Table 4). Four out of 11 (36%) of the ICF categories of body functions were identified in the ICF Core Set. The corresponding figure for activity and participation was 6 out of 9 (67%).

**Table 4:** ICF categories derived from sick leave certificates issued for long-term musculoskeletal pain ranked after relative frequency and mapped to the ICF Core Set for disability evaluation in social security. .

Sickness certificates containing the ICF category ≥ 1 time, *n* (%)*	ICF category***		ICF Core Set for disability evaluation in social security
**Body functions (*n* = 5** ** **)**			
31 (91)	b280	Sensation of pain	x
18 (53)	b710	Mobility of joint functions	x
13 (38)	b130	Energy and drive functions	
9 (26)	b140	Attention functions	
9 (26)	b455	Exercise tolerance functions	x
8 (24)	b134	Sleep functions	
5 (15)	b730	Muscle power functions	x
5 (15)	b770	Gait pattern functions	
4 (12)	b126	Temperament and personality functions	
4 (12)	b152	Emotional functions	
4 (12)	b410	Heart functions	
**Activity and participation (*n* = 14** ** **)**			
10 (29)	d430	Lifting and carrying objects	x
8 (24)	d850	Remunerative employment	
8 (24)	d410	Changing basic body position	x
8 (24)	d450	Walking	x
7 (21)	d160	Focusing attention	
7 (21)	d445	Hand and arm use	x
6 (18)	d415	Maintaining a body position	x
5 (15)	d240	Handling stress and other psychological demands	x
4 (12)	d640	Doing housework	

* Relative frequency. The number of certificates containing the ICF category divided by all sickness certificates issued for long-term musculoskeletal pain (*n *= 34).

** *n *= total number of ICF categories in the ICF Core Set per component.

*** The ICF categories are aggregated to the second level, and only those categories present in more than 10% of the sickness certificates are listed.

## 4. Discussion

This study aimed to validate the content of ICF and ICF Core Sets in relation to information on work-related disability in sick leave due to depression and long-term musculoskeletal pain. The results show that most of the concepts regarding work-related disability derived from sick leave certificates issued for depression (83%) and long-term musculoskeletal pain (75%) could be linked to second level ICF categories. The Comprehensive ICF Core Set for depression covered almost all ICF categories of the components of body functions and activity and participation, derived from the ICF linking. The corresponding figures were much lower for both the Brief ICF Core Set for depression and the ICF Core Set for disability evaluation in social security.

### 4.1. Findings in relation to other studies

#### 4.1.1. ICF-coded health data on work-related disability in sick leave certificates in relation to the content of ICF

All certificates had information linkable to ICF categories of body functions and activity and participation. Although no previous Swedish study used the full ICF linking method to map health information in sick leave certificates to ICF categories on a detailed level, the result still indicates an overall improvement in the use of such health data over the years. In a study of sick leave certificates issued in both primary care and hospitals in 2007, information on sick leave certificates was linked to a less detailed level of ICF (component level) (21). The result was that 65% of the certificates contained descriptions of functioning that could be linked to ICF, and in a follow-up two years later, the result improved to 79%. A Swiss study on certificates for work capacity evaluation explored a more detailed six-page form, issued for chronic widespread pain and low back pain, that included broader questions on contextual factors, such as environmental and personal factors, and used an ICF linking methodology on a detailed level (22). Although not fully comparable to our study, some of the findings are worth discussing. We found in our study that 83% of the information given for depression, and 75% for long-term musculoskeletal pain, was possible to link to ICF categories. These are higher ratios than in the Swiss study (53.7% for chronic widespread pain and 53% for low back pain), which might be explained by differences in the purpose of the medical evaluation and the forms used. In line with the Swiss study, we found that ICF was not detailed enough to link the full descriptions of anatomy from the certificates to ICF categories. Also, information on assessment methods, changes in health over time, and results of tests were frequent among the non-ICF-linkable information found in both studies.

#### 4.1.2. ICF-coded health data on work-related disability in sick leave certificates issued for depression, in relation to the content of ICF Core Set for depression

##### 4.1.2.1. Comprehensive ICF Core Set

The ICF categories derived from certificates regarding depression were well captured by the Comprehensive ICF Core Set for depression, although the most frequent ICF category for activity and participation, “d160 Focusing attention”, was not included in the ICF Core Set (11). The discrepancy could be explained by the methodology we used; when the information given in the certificate was insufficient for choosing one of the components, multiple codes could be assigned. This was most often applied to the concept “focusing on”, which in a majority of cases was linked to both “b140 Attention functions” (included in the ICF Core Set) and “d160 Focusing attention” (not included in the ICF Core Set).

##### 4.1.2.2. Brief ICF Core Set

We expected a high degree of conformity between the content of the Brief ICF Core Set for depression and the ICF categories derived from the certificates, but interestingly the coverage was 50% or less per component. Sleep and memory functions were described in around a third of the certificates (36% and 28%, respectively) but were not included in the Brief ICF Core Set. In the Swiss ICF linking study mentioned above, thresholds of 25% and 50% relative frequency were used to find the relevant ICF categories (22). Our sample size is rather small, and it is not possible to draw any certain conclusions about relevance as potential additions to the ICF Core Sets. Still, if a 25% threshold is applied to our material, the ICF categories of “b134 Sleep functions” (rel. frequency = 36%) and “b144 Memory functions” (rel. frequency = 28%) would be considered relevant for this context, even though they are not included in the Brief ICF Core Set for depression. Also worth mentioning is the ICF category “d710 Basic interpersonal interactions”, the fourth most common ICF category of the component of activity and participation, but below the threshold (16%). This category might also be useful in this context since interpersonal interactions are known to be important for workability (12).

#### 4.1.3. ICF-coded health data on work-related disability in sick leave certificates issued for long-term musculoskeletal pain, in relation to the content of ICF Core Set for disability evaluation in social security

The most frequent ICF categories derived from the certificates issued for patients with long-term musculoskeletal pain were well covered by the ICF Core Set for disability evaluation in social security, with a few important remarks. Difficulties connected to energy level were often mentioned in the certificates, but the corresponding ICF category “b130 Energy and drive functions” (rel. frequency 38%) is not included in the ICF Core Set. Our results indicate that this ICF category is relevant for work-related disability, and other studies point in the same direction. In a previous validation study of the Core Set, “b130 Energy and drive functions” was mentioned as one of the most frequent categories proposed to be added to the ICF Core Set (13). Also, “b130 Energy and drive functions” was the third most frequent ICF category derived from Swedish certificates for long-term sickness absence (23). The category is also included in the Brief ICF Core Set for chronic widespread pain as well as in the minimal generic ICF Core Set (24, 25). We also found that the certificates, to a high degree, contained information on sleep linked to “b134 Sleep functions” (rel. frequency 24%), a category not included in the ICF Core Set for disability evaluation in social security. This result is in line with the Swiss study of medical reports issued for chronic widespread pain (*n* = 50) and low back pain (*n* = 45), where sleep was mentioned in a majority of the analysed reports (22). On the other hand, the ICF categories “b140 Attention functions” and “d160 Focusing attention” had a high relevance according to their relative frequencies (26% and 21%) in our study, but contrary to information on sleep, information on attention was not present in the Swiss medical reports mentioned above (22).

### 4.2. Information on remunerative employment

Finally, we found information regarding employment and classified it as “d850 Remunerative employment” in 40% of the certificates issued for depression and 24% for long-term musculoskeletal pain (Tables 3, 4). However, the ICF category d850 is not included in the Brief ICF Core Set for depression nor the ICF Core Sets for disability evaluation in social security (11, 12). The meaning units linked to this category describe the person's situation in relation to work, namely if and how the work hours had been reduced, or if work tasks had been or should be changed. Since the certificate aims to capture a health condition's effect on a person's functioning in relation to work, the presence of detailed employment information is expected. However, it does not seem possible to capture the information given in the certificates on this topic in a useful way with only one ICF category.

### 4.3. Strengths and weaknesses

We consider the sample to be representative of primary care in Sweden. Since the community covers agricultural, service, and production industries, the socio-economic distribution in the region mirrors well Sweden as a whole, and the community has therefore been explored in previous studies (17, 18, 23). Information regarding disabilities in social insurance is still reported in the same way as in 2012, thus the results are considered relevant. Furthermore, the ICF experts had different skills by profession, which guaranteed the quality of the ICF linking. Also, the saturation of ICF categories was reached for both diagnoses. Considering these aspects, there might still be a risk of differences in the choice of endorsed ICF categories by other coders and a larger or national sample.

### 4.4. Implications

The results could be used to identify any lack of knowledge, i.e., blank spots in the ICF Core Sets when used in existing and future supports for documentation and assessments in the sick leave process. The knowledge gained from the study could be used in systems designed to provide the relevant ICF categories in digital coding tools to support physicians in reporting disabilities in sickness certificates. When the technology is ready to fully support more (semi-) automatised methods for ICF linking, the classification will be of importance in building up knowledge about the reasons behind sick leave in a perspective wider than that of the diagnosis. Moreover, research in this field could use the ICF framework to structure the complex data available from the sickness certification process. Using this framework, future research could investigate the effects of educational methods on physicians, study potential differences within or between countries, and make the process more effective.

## 5. Conclusions

We found that the vast majority of the health data on work-related disability in sick leave certificates could be linked to ICF categories on a detailed level, indicating that ICF is a feasible code scheme for such information. As expected, the Comprehensive ICF Core Set for depression covered the ICF categories derived from the certificates for depression to a high degree. Also, we found support for additions to the Brief Core Set for Depression (sleep, memory functions) and ICF Core Set for disability evaluation in social security (energy level, sleep and attention) when used to support the transfer of the information regarding work-related disability. The proposed additions are an interesting finding, but the need should be validated in a larger sample.

## Data Availability

The raw data supporting the conclusions of this article will be made available by the authors, without undue reservation.
